# Spiritual Needs in Patients Suffering from Fibromyalgia

**DOI:** 10.1155/2013/178547

**Published:** 2013-11-20

**Authors:** M. Offenbaecher, N. Kohls, L. L. Toussaint, C. Sigl, A. Winkelmann, R. Hieblinger, A. Walther, A. Büssing

**Affiliations:** ^1^Department of Physical Medicine and Rehabilitation, University Hospital Munich, 81377 Munich, Germany; ^2^Department of General Medicine, University Clinic IV, 80336 Munich, Germany; ^3^Generation Research Program, Human Science Center, University of Munich, 83646 Bad Tölz, Germany; ^4^Division of Integrative Health Promotion, University of Applied Sciences, 96450 Coburg, Germany; ^5^Brain, Mind & Healing Program, Samueli Institute, Alexandria, VA 22314, USA; ^6^Department of Psychology, Luther College, Decorah, IA 52101, USA; ^7^Institute for Integrative Medicine, Faculty of Medicine, Witten/Herdecke University, 58313 Herdecke, Germany

## Abstract

The objective of this study was to assess spiritual needs of patients with fibromyalgia syndrome (FMS) and to evaluate correlations with disease and health associated variables. Using a set of standardized questionnaires (i.e., Spiritual Needs Questionnaire, Fibromyalgia Impact Questionnaire, SF-36's Quality of Life, Brief Multidimensional Life Satisfaction Scale, etc.), we enrolled 141 patients (95% women, mean age 58 ± 10 years). Here, needs for *inner peace* and *giving/generativity* scored the highest, while *existential needs* and *religious needs* scored lowest. Particularly *inner peace* needs and *existential needs* correlated with different domains of reduced mental health, particularly with anxiety, the intention to escape from illness, and psychosocial restrictions. Thirty-eight percent of the patients stated needs to be forgiven and nearly half to forgive someone from their past life. Therefore, the specific spiritual needs of patients with chronic diseases should be addressed in clinical care in order to identify potential therapeutic avenues to support and stabilize their psychoemotional situation.

## 1. Introduction

The fibromyalgia syndrome (FMS) is a prevalent syndrome characterized by a variety of symptoms such as chronic pain, disturbed sleep, stiffness, fatigue, and psychological distress [1]. These symptoms have substantial impact on many domains of patients' lives. Social and leisure activities, household and outdoor activities are impaired in FMS patients [2, 3], and functional impairments were rated as incriminatory [4]. There is a complex interrelationship between the functional impairment in these areas and psychosocial factors, that is, less social support and higher depressive symptoms predict greater disability [5]; FMS patients with more functional limitations were distressed and reported higher levels of anxiety and depression [6]. The direction of these pathways is difficult to assess as it is well known that both anxiety and depression are not only associated with activity-related discomforts but also related to reduced quality of life [7]. A high proportion of FMS patients report dissatisfaction with their restrictions performing tasks, participating in social and work activities and with their ability to interact with their family and friends [4]. FMS also has a negative effect on the working ability of people on a large scale [8]. In a study by Henriksson [9], 75% reported symptoms that had negatively influenced the work situation and 69% found their work stressful; 52% of those patients who were part of the workforce reported to work shorter than prior to the FMS symptoms, 34% worked at a slower pace, and 38% needed frequent rest periods.

FMS may also affect the family setting and relationships with friends. Marcus et al. [10] reported that 50% of their patients admitted that FMS had mildly to moderately damaged relationship(s) with their spouse(s)/partner(s) or contributed to a break-up with a spouse or partner, and 50% reported to be not satisfied with their current spouse/partner relationship. Preece and Sandberg [11] found that FMS patients' self reported family stressors, strains, and distress were significantly associated with an increase in health problems as well as functional disability.

In a recent qualitative study [12], FMS patients explained their phenomenal experience related to their suffering as a course of a “giant mess” of unpleasant symptoms, some of which were understood to be symptoms of FMS and some of which were the interactive or parallel effects of comorbid illness. The participants in this study stated that they had to spend considerable efforts at imposing order and sense on complexity and multiplicity, in terms of the instability of their symptoms.

To sum up, FMS patients are challenged to cope, on a wide range, with symptoms and disease consequences affecting many domains of their lives. In fact, patients with chronic pain use a number of cognitive and behavioural strategies to cope with their pain, including spiritual/religious forms of coping, such as prayer, and seeking spiritual support to manage their pain [13]. Studies have shown that spirituality/religiosity (SpR) as a coping strategy was associated with positive affect (mood), but not with pain specific outcomes, while negative religious coping strategies were not associated with any of the outcomes [14].

SpR was identified as a relevant resource to cope even among more secular German patients with chronic pain diseases. Interestingly, SpR was associated with positive disease interpretations such as challenge and value, but not with negative interpretations of disease, while life satisfaction or depressive escape from illness was not significantly associated with measures of SpR [15]. In fact, the increasing utilization of SpR goes far beyond fatalistic acceptance, but can be regarded as an active coping process, even in a secular society [15].

Meanwhile, studies have clearly shown that several patients with chronic diseases have unmet spiritual needs [16]. These needs can be referred to the categories connection/relatedness, meaning/purpose, peace, and transcendence [16]. Balboni et al. [17] found that 72% of US American patients with advanced cancer reported that their spiritual needs were supported minimally or not at all by the medical system, while 47% felt supported minimally or not at all by a religious community, too. Among German patients with chronic pain diseases (predominantly chronic pain diseases and also cancer), several unmet spiritual needs were identified, and these refer to inner peace needs and generative relatedness on a personal level, whereas needs related to transcendent relatedness were of minor relevance [18, 19]. Using the same measure, a similar pattern was found also in patients with chronic diseases (predominantly cancer) from Shanghai [20]. However, in healthy elderly living in residential/nursing homes all these needs scored very low, particularly religious and existential needs, while inner peace needs were of some relevance and needs for giving/generativity were of highest relevance [21]. Thus, one may suggest that spiritual needs related to the categories of relatedness and peace are of higher relevance than transcendent and existential issues—at least in secular societies.

While one may expect that spiritual needs are stated as a result of an experienced lack or loss, it is of interest that particularly existential needs and needs for inner peace were inversely related to spiritual well-being (which indicates a lack or loss), while in contrast religious needs were positively associated (which would indicate that a religious attitude might be a prerequisite to express such needs) [19].

The objective of our study was to assess which spiritual needs were prevalent in a specific and circumscribed sample of German patients with FMS and to evaluate associations between these needs and disease associated variables, mental health associated variables (i.e., anxiety, depression, loneliness, etc.), and measures of life satisfaction and health related quality of life.

## 2. Patients and Methods

### 2.1. Participants

A questionnaire battery consisting of the measurement instruments described below was sent to 300 patients, who had been treated in a multidisciplinary rehabilitation program at the department of Physical Medicine and Rehabilitation between 2004 and 2008. This department is a tertiary care center located at the University Hospital in Munich. All patients fulfilled the American College of Rheumatology (ACR) criteria for FMS [1] at the time of inception to the treatment program.

### 2.2. Self Report Variables

The following sociodemographic variables were collected: marital status, age, level of education, religious orientation. Patients' religious/spiritual self-categorization was measured with two items derived from the SpREUK questionnaire (SpREUK is an acronym of the German translation of “Spiritual and Religious Attitudes in Dealing with Illness”), that is, f1.1. (“To my mind I am a spiritual individual”) and f2.1. (“To my mind I am a religious individual”) [22]. Thus, we can categorize patients who regard themselves as both religious and spiritual (R+S+), religious but not spiritual (R+S−), not religious but spiritual (R−S+), or neither religious nor spiritual (R−S−).

Data on duration and intensity of symptoms indicative of FMS was collected too. Pain was assessed with a visual analog scale (VAS) of current pain severity, the frequency of severe pain during the last three months, a VAS of pain severity during the last three months, and a Tender Point Score (TPS). The TPS consists of a body image illustrating 24 regions on the back and front, which are commonly indicated as painful by FMS patients. Patients indicate pain intensity by themselves in 24 regions ranging from 0 (no pain) to 5 (extreme pain); the maximum score is 120. Due to missing items (four missing items were acceptable), the TPS was not calculated in 22 patients.

The questionnaire set included the following measures.

#### 2.2.1. Spiritual Needs Questionnaire (SpNQ)

To measure patients' spiritual needs, we used the Spiritual Needs Questionnaire (SpNQ) [18, 23] which can be used either as a “diagnostic tool” with 30 items or as a measure with 19 or 20 items which were assigned to four main factors [23].

1. *Religious needs*, that is, praying for and with others, praying alone, participating in a religious ceremony, reading spiritual/religious books, turning to a higher presence (i.e., God, angels).
2. *Existential needs* (Reflection/Meaning), that is, reflecting on one's life, talking with someone about the meaning of life/suffering, dissolving open aspects in life, talking about the possibility of life after death, and so forth.
3. Need for *inner peace*, that is, wish to dwell at places of quietness and peace, plunge into the beauty of nature, finding inner peace, talking with others about fears and worries, being admired by others.
4. Need for *giving/generativity*, that is, actively and autonomous intention to solace someone, passing along one's own life experiences to others, and to be assured that your life was meaningful and of value.

Patients rate whether they currently have the respective needs (yes/no) and how strong they were to them. The self-ascribed importance was measured on a 4-point scale from disagreement to agreement (0—not at all; 1—somewhat; 2—very; 3—extremely). For all analyses, we used the mean scores of the respective scales described above; the higher the scores, the stronger the respective needs are.

Because some patients did not respond to all items of the respective scales, the mean scores were calculated only when 2/3 or 3/5 of items were present. Thus, the SpNQ scales were not calculated in 6 and 8 persons, respectively.

#### 2.2.2. SpR Attitudes and Convictions in Coping with Chronic Diseases (SpREUK-15)

The SpREUK-15 questionnaire measures SpR attitudes and convictions of patients dealing with chronic diseases [22, 24]. It differentiates three factors [22, 24].

1. *Search* scale, or search (for support/access to SpR), deals with patients' intention to find or have access to a spiritual or religious resource which may be beneficial for coping with illness and with their interest in spiritual or religious issues (insight and renewed interest), and so forth.
2. *Trust* scale, or trust (in higher guidance/source), is a measure of intrinsic religiosity; the factor deals with patients' conviction that they want to be connected with a higher source and with their desire to be sheltered and guided by that source, whatever may happen to them, and so forth.
3. *Reflection* scale, or reflection (positive interpretation of disease), deals with patients' cognitive reappraisal of life because of illness and subsequent attempts to change (i.e., reflecting on what is essential in life, to change aspects of life or behavior, looking for opportunities for development, believing that the illness has meaning, etc.).

The SpREUK-15 scores items on a 5-point scale from disagreement to agreement (0—does not apply at all; 1—does not truly apply; 2—do not know (neither yes nor no); 3—applies quite a bit; 4—applies very much). The scores were referred to a 100% level (transformed scale score). Scores >50% indicate higher agreement (positive attitude), while scores <50% indicate disagreement (negative attitude).

The mean scores were calculated only when 3/5 of items were present; thus these scores were not calculated in 4 to 5 persons.

#### 2.2.3. Hospital Anxiety and Depression Scale

The Hospital Anxiety and Depression Scale (HADS) is a brief self-report questionnaire measuring anxiety (7 items) and depression (7 items) [25]. Each item is rated on a scale from 0 to 3, giving a possible score of 21 for anxiety and depression. For either subscales a score of 11 or higher is indicative of a present mood disorder [26]. Due to missing items, the two HADS subscales were not calculated for one patient.

#### 2.2.4. Fibromyalgia Impact Questionnaire (FIQ)

The FIQ is an assessment and evaluation instrument developed to measure fibromyalgia patients' status, progress, and outcomes. It has been designed to measure the components of health status that are believed to be most affected by fibromyalgia [27]. A total score of the FIQ is calculated on the basis of physical functioning, number of days felt good, pain, fatigue, morning tiredness, stiffness, anxiety, and depression ranging from 0 to 80, with 80 indicating maximum fibromyalgia impact. Due to missing data, the FIQ score was not calculated in 17 patients.

#### 2.2.5. Quality of Life Scale (QOLS)

The QOLS is a 16-item questionnaire adapted by Burckhardt et al. [28] for the use in chronic disease patients, including patients with fibromyalgia [29]. Items are rated on a 7-point Likert scale, whereas higher values denote higher quality of life. Here, the score was calculated for all patients.

#### 2.2.6. Short Form 36 (SF-36)

The SF-36 is a 36-item instrument for measuring health status and outcomes from the patient's point of view and has been translated and validated into numerous languages including German [30]. The SF-36 measures the following eight health concepts: limitation in physical activities, limitation in usual role activities, bodily pain, general health perception, vitality (energy and fatigue), limitation in social activities, limitations in usual role activities because of emotional problems, mental health (psychological distress and well-being). Higher values indicate, for example, less limitation, more pain or higher vitality, and so forth. Due to missing data, with respect to the subscales, for up to 3 patients the scores were not calculated; here the two main components (physical and mental health) were not calculated in 9 cases.

#### 2.2.7. Escape from Illness Scale

A depressive intention to run away from the current situation might be an indicator of a patient's struggle with disease and associated with psychosocial and spiritual needs. The three-item scale “Escape” is an indicator of such an escape-avoidance strategy addressing an attitude of fearful insecurity, a tendency to run away from illness, and the wish that all this could have been nothing more than a bad dream (i.e., “fear of what illness will bring,” “would like to run away from illness,” “when I wake up, I don't know how to face the day”) [31]. In a study involving patients with depressive disorders, we demonstrated that this “Escape” scale correlated strongly with depression, with disease perceptions (appraisals) such as “weakness/failure,” and “punishment,” and negatively with life satisfaction [32, 33].

The items were scored on a 5-point scale from disagreement to agreement. For all analyses, we used the mean scores of the “Escape” scale based on a scale of 100%. Scores >50% indicate the presence of this attitude, and scores <50% represent a lack of this attitude. The mean scores were calculated only when 2/3 of items were present factor; here the “Escape” scores were not calculated in 24 persons.

#### 2.2.8. Brief Multidimensional Illness Scale (BMLSS)

Life satisfaction was measured using the Brief Multidimensional Life Satisfaction Scale (BMLSS) [34]. The items address intrinsic (i.e., myself, life in general), social (i.e., friendships, family life), external (i.e., work situation, where I live) and prospective (i.e., financial situation, future prospects) dimensions of life satisfaction as a multifaceted construct. The internal consistency of the instrument was found to be good in the validation study [34]. This current study used the 10-item version, which includes satisfaction with one's health condition and ability to deal with daily concerns about life (BMLSS-10).

Each of these 10 items was introduced by the phrase “I would describe my level of satisfaction as …”, and was scored on a 7-point scale ranging from dissatisfaction to satisfaction (0—terrible; 1—unhappy; 2—mostly dissatisfied; 3—mixed (about equally satisfied and dissatisfied); 4—mostly satisfied; 5—pleased; 6—delighted). The BMLSS-10 mean score was based on a scale of 100% (“delighted”). Scores >50% indicate higher life satisfaction, while scores <50% indicate dissatisfaction.

The mean scores were only calculated when 7/10 of items were present. The BMLSS-10 scores were not calculated in 5 persons.

#### 2.2.9. UCLA Loneliness Scale

The UCLA loneliness scale is a 20-item questionnaire measuring general feelings of social isolation, loneliness, and dissatisfaction with one's social interactions [35]. The 20 items are rated on a 5-point Likert scale ranging from totally disagree [1] to totally agree [5]. Scores on the scale range from 20 to 100 with higher scores indicating more loneliness. The scale was not calculated for 2 patients.

#### 2.2.10. Catastrophizing Subscale of the Coping Strategy Questionnaire (CSQ)

We further administered the catastrophizing subscale of the Coping Strategy Questionnaire (CSQ-catastrophizing) [36]. The CSQ is an instrument that has been used in patients with chronic pain conditions including FMS [37]. The CSQ contains 8 subscales that assess cognitive and behavioural pain coping mechanisms as well as 2 items measuring perceived effectiveness of strategies for controlling and decreasing pain. In our study, we only administered the subscale catastrophizing of the CSQ. Due to missing items (two missings were allowed), the scale was not calculated for 3 patients.

## 3. Statistical Analysis

SPSS 21.0 was used for the statistical analyses. Missing data was not replaced. Thus, some scales were not calculated for all patients. In a first step of analysis, descriptive analyses (mean, median, and standard deviation) were computed for scales and subscales. Associations between scales were analyzed on the basis of first order correlations (Pearson's *r*). In a further step, stepwise multiple regression analyses were used to indentify predictors for spiritual needs. *P* was set to *P* < 0.05, given the exploratory character of the study. With respect to the correlation analyses, we regarded *r* > 0.50 as a strong correlation, an *r* between 0.30 and 0.50 as a moderate correlation, an *r* between 0.20 and 0.30 as a weak correlation, and *r* < 0.20 as no or negligible correlation.

## 4. Results

141 out of 300 patients sent the questionnaires back to the department (response rate = 47%). Sociodemographic variables and other variables are depicted in Table 1. Of importance is the fact that the patients analyzed herein exhibited a high percentage of clinical relevant anxiety (58% of patients with scores greater than 8) and/or depression (44% of patients with scores greater than 8). In Table 2 the prevalences of selected needs as well as the means, medians, and standard deviations of the subscales of the SpNQ are displayed.

### 4.1. Spiritual Needs Scores in the Sample

As shown in Table 3, *religious needs* scored significantly higher in patients with a religious attitude (R+S+ and R+S−) and lowest in R−S− patients, while *existential needs* were significantly higher in spiritual individuals (R+S+ and R−S+) and low in R−S−. *inner peace needs* and *giving/generativity needs* were highest in R−S+, although these differences were not statistically significant when compared to the other self-categorization groups.

These scales did not significantly differ with respect to family status (data not shown); the educational level had a significant influence only in trend on the *religious needs*, which were highest in those with a lower educational level (*F* = 2.3; *P* = 0.080). Due to the fact that only 7 men were in the sample, we cannot state any statistically significant effects with respect to gender.

### 4.2. Correlations between Spiritual Needs and Health Related Variables

Correlation analyses revealed that the spiritual needs were intercorrelated (Table 4), particularly *inner peace* needs correlated strongly with *existential needs*. Both pain scores (tender point score and fibromyalgia impairment) and physical health (SF-36) were either not or just weakly associated with the respective spiritual needs—only *inner peace* needs were moderately associated with fibromyalgia impact (Table 4).

*Religious needs* correlated strongly with religious Trust, and moderately with spiritual Search, but with none of the health related variables. 

*Existential needs* were moderately associated with the SpREUK scales, with anxiety, escape, and inversely with SF-36's mental health scores.

*Inner Peace needs* were moderately associated with anxiety, escape, depression catastrophizing, reduced mental health, and SpREUK's reflection scale. 

*Giving/generativity needs* were only weakly associated with the respective measures and subscales, best with bodily pain (*r* = −0.26).

### 4.3. Predictors of Spiritual Needs

Stepwise multiple regression analyses were used for identifying the most significant predictors (Table 4). The variables which were empirically found to be intercorrelated with spiritual needs included SpREUK's Search, Trust, and Reflection scales, Fibromyalgia Impact scores, anxiety and depression, Escape, loneliness, catastrophizing, life satisfaction, and the SF-36's mental health component. 

As shown in Table 5, *religious needs* were predicted best (*R* ^2^ = 0.45) by religious Trust, with a further positive influence of spiritual Search, fibromyalgia impairment scores, and life satisfaction.

*Existential needs* were predicted best (*R* ^2^ = 0.43) by anxiety, with a further impact of spiritual Search and religious Trust. 

*Inner peace needs* were predicted best (*R* ^2^ = 0.29) by anxiety, and further by the ability to reflect in terms of a positive interpretation of disease. 

*Giving/generativity needs* were explained by several variables (*R* ^2^ = 0.25), best by low depression scores, and a further positive influence of, however, catastrophizing as a coping strategy, the ability to reflect, and reduced life satisfaction.

### 4.4. Additional Findings

Semantically similar items, which are not specifically related to SpR but might potentially be of importance for the interpretation of data were collapsed into the following sum scores (Table 2): (1) scale “need for participating” included items N28 (“being more involved in family business”), N29 (“being invited by friends”), and N25 (“being more connected with own family”); (2) scale “need for attention/support” included N1 (“receiving greater care from others”) and N30 (“receiving more support from own family”); (3) scale “need for forgiveness” included N16 (“forgive someone from past life”) and item N17 (“to be forgiven”). It is important to note that these “sum scores”, from a psychometric point of view, are not considered to represent reliable factors of questionnaire instruments, but they are potentially helpful for exploring further associations.  Table 6 shows the correlations of the three scales with the other variables, scales, and subscales.

## 5. Discussion

This study specifically enrolling patients with fibromyalgia confirms previous findings among patients with various chronic pain diseases [19] that secular needs for *inner peace* and *giving/generativity* scored higher than *religious needs* or *existential needs*. In a recent study we investigated the spiritual needs of 392 patients (67% women, mean age 56 ± 14 years; 61% Christian denomination) with chronic pain diseases (86%) and cancer (14%) [19], while in the current study patients with fibromyalgia (95% women, mean age of 58 ± 10 years; 73% Christians) were enrolled. The patients in this study exhibited similar sociodemographic and disease characteristics as well as nonhealth and health related quality of life compared to other FMS samples [29, 38, 39]. In contrast to the sample of various chronic pain diseases and cancer [19], FMS patients' needs scored somewhat higher. One may suggest that the dominance of women in this study (95%) compared to the previous study (67% women) might explain these slightly higher needs scores because the female gender is known to be associated with higher scores of spiritual needs [19].

Moreover, in this study, *existential needs* and *inner peace* needs were correlated moderately with the Escape scale, which was associated in the later study only weakly with *existential needs*. Similarly to the former study, pain intensity or affections itself had no relevant influence on the needs expressed.

Interestingly, particularly the strongly interconnected factors *existential needs* and *inner peace *needs were moderately associated with anxiety, Escape, and reduced mental health, but not with physical health, and only weakly with fibromyalgia associated impairment. In contrast, *religious needs* and also *giving/generativity* were not significantly associated with health associated variables. This suggests that *religious needs* may be expressed because of a reliance on religiosity as a resource to deal with life concerns, as a matter of trust in God who carries through, and not necessarily because of an impaired life satisfaction. In fact, religious Trust was identified in this study as the best predictor of *Religious needs*, with a further impact of spiritual Search, fibromyalgia associated impairment, and life satisfaction.

Also in this study, anxiety was the best predictor of *Existential needs* and *Inner Peace* needs. Interestingly, spiritual Search and religious Trust were contributing variables to *existential needs*, while for *inner peace needs* only the ability to reflect in terms of a positive interpretation of illness was a further contribution variable. This specific pattern is plausible from a theoretical point of view because reflecting previous life, finding meaning in illness and suffering, talking with someone about the question of meaning in life and the possibility of life after death, and also to forgive someone from the past life are existential needs which may have a spiritual or even religious connotation. In contrast, to immerse in the beauty of nature, to dwell at quiet and peaceful places, finding inner peace, and having a loving attitude toward others on the one hand, and talking about fears and worries and trying to dissolve open aspects of life on the other hand, are needs which may result in states of inner peace and release, but they are not necessarily religious issues.

The dependent variable *giving/generativity* was predicted best by low depressive symptoms, and also by the ability to reflect on what is essential in life, how to change attitudes and behaviour, and by other variables. This factor clearly points to patients' intention to be assured that life is meaningful and of value, that one is nevertheless able to solace someone, and able to pass along one's own life experiences to others. Here the dimension of generative relatedness is connected with an existential, meaning making issue.

### 5.1. Interpretation of Additional Topics: Forgiveness

Forgiveness can be both a secular existential istic and a religious issue, depending on the individual context. As a result of such processes of forgiveness, inner peace states may occur. Nearly half of the patients in our sample had the need to forgive someone from their past life, and 38% to be forgiven. Having these needs was weakly associated with anxiety and negatively with SF-36's mental health component. Although open conflicts in life which may require forgiveness can be associated with mental health affections, we cannot draw any causal conclusions. Longitudinal studies addressing this issue are required.

Nevertheless, these findings fit to the model proposed by Toussaint et al. [40] that in patients with fibromyalgia forgiveness can serve as a crucial coping resource that may have direct effects on mental and physical health, as well as effects on health through affective control mechanisms and control of central sensitization and dysregulation. Research indicates that forgiveness may have soothing effects on the sympathetic nervous system (e.g., [41], and may offer some potential benefits for assuaging the damaging effects of stress hormones such as cortisol [42–44]). Furthermore, the findings confirm patient benefits from forgiveness in other patient populations [45–49].

Moreover, these results show that FMS patients—like other patients with chronic diseases, too—may have prevalent needs to forgive and be forgiven, and this is consistent with the only other known study of forgiveness needs in chronic illness. In his study, Barry [50] found that when cancer patients were asked during a new patient orientation program about their need to forgive a prior healthcare professional, God, themselves, or others, 39% reported forgiveness issues that needed to be addressed and half of these people reported high to severe forgiveness concerns. In a second study that used an anonymous response format, the percentage of patients reporting forgiveness issues rose to 61% and approximately half of those reported high to severe forgiveness concerns. Taken together, the data from the present study and that of Barry's suggest that forgiveness needs in chronic illness are surprisingly common, and that forgiveness needs in FMS patients may be similar to those in cancer patients. Barry's work suggests [50] that unfortunately patients are reluctant to communicate these needs to their healthcare providers; one may assume a similar effect also for patients with chronic pain diseases. On the other hand, an alternative explanation refers to cancer patients' experience that their life time might be limited, and thus existential issues are of higher importance to them when compared to patients with chronic pain diseases [18] who often have to learn to live with their disease. Nevertheless, given the high needs of patients in the realm of forgiveness, it behoves medical professionals to become better adept and inquiring about these needs and offering appropriate education, intervention, or referral. Several options for psychoeducational forgiveness training exist (see [40]) and methods specifically tailored to healthcare settings are being developed [50].

### 5.2. Interpretation of Additional Topics: Participation and Support

More than half of the patients indicated a need for more participation in social activities and support by family and others. Particularly the needs for attention and support were moderately associated with a variety of disease as well as psychosocial variables, including anxiety and depression, loneliness, and daily life affections through the pain. According to Eisenberger and Lieberman [51] there is increasing evidence from animal and human neuroimaging studies suggesting that physical and social pain overlap in their underlying neural circuitry and computational processes. It has also been suggested that the social-attachment system borrowed the computations of the pain system to prevent the potentially harmful consequences of social separation. Eisenberger and Cole [52] even made the point that it is possible that long-term experiences of social disconnection (loneliness) or connection (social support) may fundamentally alter the function and connectivity of the neural systems, consequently affecting how they relate to health relevant physiological outputs.

In qualitative studies, FMS patients repeatedly report that being stigmatized was an outstanding theme [53, 54]. They described perceptions of being left alone with their illness, due to a lack of understanding and acceptance from others. As a result of stigmatization, FMS patients withdraw from several areas of social life [55], further enhancing the feeling of being worthless and of loneliness. Therefore, it is conceivable that FMS patients express a need to participate and be cared for and this need to be more connected with others is also underpinned by the high prevalence of the needs to turn to someone in a loving attitude and to solace someone in our sample. Feeling connected, giving and receiving support is an important part of human life and it has recently been demonstrated that individuals who interact more with close, supportive, and comforting individuals on a daily basis show reduced neurocognitive and physiological stress reactivity to a social stressor [56]. The authors of that study concluded that this reduction in stress responses, over time, may result in better health outcomes.

### 5.3. Limitations

Some limitations of our study need to be addressed. The FMS patients were recruited in a tertiary referral center and all of them participated in the past in a multidisciplinary treatment program which also included elements of psychosocial education and emotion control. The response rate in our study was 46%; we are not able to compare the responders to the nonresponders. Of course one cannot exclude the possibility that particularly those who have no interest in spiritual/religious issues may have not responded to the questionnaires, and thus our results might be too positive for religious patients. However, 59% of the patients would not regard themselves as religious—and this amount is consistent with previous findings among patients with chronic pain diseases [15, 19]. Finally, our patient group had a long history of FMS related symptoms and high levels of pain and other symptoms as well as functional limitations. Therefore, the question remains whether we would find similar results in nonpatients (i.e., FMS patients in the population who have not sought health care). However, when compared to data of a previous study among patients with chronic pain conditions, the pattern and level of spiritual needs were similar [19].

### 5.4. Outlook

Clearly there are high proportions of FMS patients who have specific spiritual needs. But where are those met? The current health care system is based on the biomedical model which has changed the focus of medicine from a caring, service-oriented model to a technological, cure-oriented model [57].

There are several studies showing that emotional, social, and spiritual issues in the doctor-patient encounter are often not addressed and/or discussed [58–61]. Ellis et al. [62] performed a qualitative study with family physicians and identified several barriers to spiritual assessment, including a physician's upbringing and culture, lack of spiritual inclination or awareness, resistance to exposing personal beliefs, and belief that spiritual discussions will not influence patients' illnesses or lives. The participants also postulated patient barriers, including fears that their physician might judge them for their spiritual views or consider their raising spiritual questions inappropriate. Further barriers were indicated in a recent investigation by Vermandere et al. [63] such as lack of formal training and appropriate strategies as well as lack of time. Many physicians in that study saw it as their role to identify and assess patients' spiritual needs, despite perceived barriers. However, they struggled with spiritual language and experienced feelings of discomfort and fear that patients will refuse to engage in the discussion. The latter seems to be incomprehensible insofar that the result of our study and the results of others [64–66] indicate that a great proportion of patients have a great need for spiritual issues to be addressed in the medical encounter.

However, there is also growing interest in medicine to include spiritual or compassionate care in order to serve the whole person—the physical, emotional, social, and spiritual. Family physicians view spirituality as a significant dimension of human experience that embraces sustaining and enlivening relationships with spirit and the pursuit and expression of meaning and purpose [67]. The World Health Organization already reported 1998 (quoted in [68]): “Until recently the health professions have largely followed a medical model, which seeks to treat patients by focusing on medicines and surgery, and gives less importance to beliefs and to faith in healing, in the physician and in the doctor­patient relationship. This reductionist or mechanistic view of patients is no longer satisfactory. Patients and physicians have begun to realize the value of elements such as faith, hope, and compassion in the healing process.” Larry Culliford, a UK psychiatrist, summarized it as follows [68]: “Many see religion and medicine as peripheral to each other, yet spirituality and clinical care belong together. The time is thus ripening for doctors to recall, reinterpret, and reclaim our profession's sacred dimension.” And we would like to add: “… in order to recognize and to address our patients' spiritual needs”.

## 6. Conclusion

Evidently, a high proportion of FMS patients indicated specific spiritual needs in different domains which were associated particularly with anxiety and specific psychosocial restrictions. Therefore, these needs should be addressed in clinical care in order to identify potential therapeutic avenues to support patients' coping with illness.

## Figures and Tables

**Table 1:** Sociodemographic and other characteristics of 141 FMS patients.

Variables	
Gender (%)	
Female	95
Male	5
Age in years (mean ± SD)	57.8 ± 10.2
Disease duration (mean ± SD)	14.1 ± 10.1
Living status (%)	
Living with a partner (married or not)	72.5
Living alone (single, divorced, widowed)	27.5
Educational level (%)	
Low (primary school)/none	42.1
Medium (secondary school equivalent)	36.1
High (high school)	12.0
Other	9.8
Religious orientation (%)	
Christian	73.3
Other	6.0
None	20.7
Spiritual/religious self-categorization (%)	
R+S+ religious and spiritual	18.2
R+S− religious but not spiritual	22.7
R−S+ not religious but spiritual	19.7
R−S− neither religious nor spiritual	39.4

**Table 2:** Prevalence of needs, mean/median, and standard deviation of subscales as well as frequency of selected items of the SpNQ.

	Prevalence of need (%)		Mean/median of subscales [range 0–3]	SD of sub scale
	No	Yes		
Subscales and items				

Religious needs			**0.74/0.50**	**0.77**
N18 pray with someone	82	18		
N19 someone prays for you	75	25		
N20 pray for yourself	42	58		
N21 participate at a religious ceremony	63	37		
N22 read religious/spiritual books	63	31		
N23 turn to a higher presence	49	51		
Need for inner peace			**1.87/2.00**	**0.76**
N2 talk about fears and worries	27	73		
N5 dissolve open aspects of your life**	38	62		
N6 immerse in the beauty of nature	12	88		
N7 dwell at quiet and peaceful places	13	87		
N8 find inner peace	21	79		
N13 have a loving attitude toward others	21	79		
Existentialistic needs (Reflection/Meaning)			**0.89/0.80**	**0.74**
N4 reflect your previous life	40	60		
N10 find meaning in illness and/or suffering	60	40		
N11 talk with someone about the question of meaning in life	58	42		
N12 talk with someone about the possibility of life after death	68	32		
N16 forgive someone from past life	53	47		
Actively Giving			**1.57/1.67**	**0.92**
N15 solace someone	21	79		
N26 pass own life experiences to others	31	69		
N27 know that your life was meaningful and of value	27	73		

Additional items				

Need for participating*			**1.39/1.33**	**0.89**
N28 being more involved in family business	51	49		
N29 being invited by friends	45	55		
N25 being more connected with own family	18	82		
Need for attention/support*			**1.15/1.00**	**1.00**
N1 receiving greater care from others	48	52		
N30 receiving more support from own family	45	55		
Need for forgiveness*			**0.88/0.50**	**1.00**
N17 be forgiven	62	38		
N16 forgive someone from past life	53	47		
N3 being taken care of someone from your community	92	8		
N24 being whole and restored	21	79		
N13 turn to someone in a loving attitude	42	58		
N14 give away something from yourself	23	77		

Prevalence of specific needs follows the self-ascribed yes/no statement (% of the respondents).

*Some items were semantically combined to nonvalidated scales.

**Item was originally part of the scale and was used here again.

**Table 3:** Spiritual needs and spiritual/religious self-categorization.

		Religious needs	Existential needs	Inner peace needs	Giving/generativity needs
All individuals	mean	0.72	0.88	1.87	1.57
	SD	0.75	0.74	0.75	0.93

R+S+	mean	1.23	1.20	1.84	1.46
	SD	0.79	0.93	0.82	0.93
R+S−	mean	1.07	0.77	1.62	1.62
	SD	0.79	0.65	0.74	0.95
R−S+	mean	0.78	1.22	2.14	1.85
	SD	0.72	0.76	0.62	0.82
R−S−	mean	0.25	0.63	1.90	1.43
	SD	0.36	0.55	0.75	0.96

*F* value		17.2	6.0	2.3	1.3
*P* value		<0.0001	0.001	0.085	n.s.

Data refer to 127 to 130 responding patients.

**Table 4:** Correlation of needs with measures and subscales.

	Religious needs	Existential needs	Inner peace needs	Giving/generativity needs
Spiritual Needs				
Religious	1	0.459**	0.235**	0.402**
Existential		1	0.542**	0.429**
Inner Peace			1	0.458**
Giving/generativity				1
SpREUK Subscales				
(spiritual) Search	0.491**	0.476**	0.217	0.165
(religious) Trust	0.570**	0.387**	0.113	0.238**
Reflection (positive interpretation of illness)	0.113	0.339**	0.312**	0.230**
Fibromyalgia Impact (FIQ)	0.138	0.228	0.297**	0.234
Tender point count	0.129	0.071	0.115	0.152
Anxiety (HADS)	0.055	0.350**	0.473**	0.217
Depression (HADS)	−0.076	0.238**	0.300**	−0.030
Escape from illness	0.029	0.294**	0.412**	0.194
Loneliness (UCLA)	0.135	−0.181	−0.234**	0.009
Catastrophizing (CSQ)	−0.003	0.287**	0.359**	0.219
Quality of life				
Life satisfaction (BMLSS-10)	0.086	−0.138	−0.263**	−0.037
Quality of life scale (QOLS)	−0.007	−0.207	−0.267**	0.005
SF-36 Physical score	−0.160	0.046	0.065	−0.155
SF-36 Mental sum score	0.000	−0.334**	−0.430**	−0.103
SF-36 Physical functioning index	−0.125	−0.025	−0.013	−0.138
SF-36 Role-physical index	−0.108	0.040	−0.151	−0.129
SF-36 Bodily pain	−0.112	−0.056	−0.150	−0.258**
SF-36 General health perceptions index	−0.102	−0.335**	−0.198*	−0.120
SF-36 Vitality	−0.078	−0.122	−0.224**	−0.080
SF-36 Social functioning	−0.024	−0.192	−0.322**	−0.083
SF-36 Emotional role	0.042	−0.239**	−0.316**	−0.130
SF-36 Mental health	−0.081	−0.315**	−0.423**	−0.174*

**P < 0.01 (Pearson; 2-tailed); significant correlations with *r* > 0.30 were bold.

**Table 5:** Regression analyses with spiritual needs as dependent variables (stepwise method).

	Beta	*T*	*P*	Collinearity statistics*	
				Tolerance	VIF
Dependent variable: religious needs (R^2^ = 0.45)					
Model 4					
(constant)		−3.086	0.003		
Trust (SpREUK)	0.442	4.643	0.000	0.693	1.443
Search (SpREUK)	0.282	3.004	0.003	0.711	1.407
Fibromyalgia impairment (FIQ)	0.372	3.560	0.001	0.576	1.736
Life satisfaction (BMLSS-10)	0.269	2.601	0.011	0.589	1.696

Dependent variable: existential needs: reflection/meaning (R^2^ = 0.43)					
Model 3					
(constant)		−1.066	0.289		
Search (SpREUK)	0.339	3.514	0.001	0.686	1.458
Anxiety (HADS)	0.415	5.013	0.000	0.932	1.073
Trust (SpREUK)	0.251	2.568	0.012	0.670	1.493

Dependent variable: peace needs (R^2^ = 0.29)					
Model 2					
(constant)		5.500	0.000		
Anxiety (HADS)	0.446	4.940	0.000	0.965	1.036
Reflection (SpREUK)	0.234	2.592	0.011	0.965	1.036

Dependent variable: Giving/Generativity (R^2^ = 0.25)					
Model 5					
(constant)		3.257	0.002		
Reflection (SpREUK)	0.285	2.941	0.004	0.914	1.094
Catastrophizing (CSQ**)**	0.319	2.349	0.021	0.467	2.143
HADS Depression (HADS)	−0.682	−3.915	0.000	0.283	3.533
Life satisfaction (BMLSS-10)	−0.310	−2.310	0.023	0.477	2.095
HADS anxiety (HADS**)**	0.290	2.002	0.048	0.411	2.436

*Because the regression coefficients may be compromised by collinearity, we checked the Variance Inflation Factor (VIF) as an indicator for collinearity. VIF > 10 is indicative of high collinearity.

**Table 6:** Correlation of needs with measures and additional subscales.

Scales and subscales	Need for participating	Need for attention/support	Need for forgiveness
Pain and mental health			
Fibromyalgia impact (FIQ)	0.334**	0.342**	0.154
Tender point count	0.248**	0.223	0.043
HADS anxiety	0.243**	0.434**	0.257**
HADS depression	0.159	0.379**	0.126
Escape from illness	0.201	0.338**	0.112
UCLA loneliness scale	−0.007	−0.340**	−0.100
CSQ-catastrophizing	0.308**	0.395**	0.159
Quality of life			
Life satisfaction (BMLSS-10)	−0.065	−0.280**	−0.075
Quality of life scale (QOLS)	−0.145	−0.339**	−0.139
SF-36 Physical sum score	−0.153	0.001	0.084
SF-36 Mental sum score	−0.234**	−0.501**	−0.223
SF-36 Physical functioning	−0.152	−0.102	0.032
SF-36 Role physical	−0.177	−0.225	0.048
SF-36 Bodily pain	−0.304**	−0.310**	−0.050
SF-36 General health perception	−0.271**	−0.218	−0.235**
SF-36 Vitality	−0.286**	−0.270**	−0.034
SF-36 Social functioning	−0.144	−0.340**	−0.163
SF-36 Emotional role	−0.203	−0.445**	−0.212
SF-36 Mental health	−0.282**	−0.481**	−0.168

Significant correlations with *r* > 0.30 were bold.

**P < 0.01 (Pearson).

TPC: tender point count. VAS: visual analogue scale; QOLS: quality of life scale; FIQ: fibromyalgia impact questionnaire.
